# Supplementary material for: Benchmarking methods for computing local sensitivities in ordinary differential equation models at dynamic and steady states
Source: PLoS One. 2024 Oct 23;19(10):e0312148. doi: 10.1371/journal.pone.0312148 (PMC11498742; doi:10.1371/journal.pone.0312148)
Supplement: S1 Text — (PDF) [file pone.0312148.s001.pdf]

S1 Text. Supplementary Information to Benchmarking methods for computing local sensitivities in ordinary differential equation models at dynamic and steady states

Polina Lakrisenko[1,3], Dilan Pathirana[2], Daniel Weindl[1,2], Jan Hasenauer[1,2*]

1 Computational Health Center, Helmholtz Zentrum München Deutsches Forschungszentrum für Gesundheit und Umwelt (GmbH), 85764 Neuherberg, Germany
2 Faculty of Mathematics and Natural Sciences, and the Life and Medical Sciences Institute (LIMES), Rheinische Friedrich-Wilhelms-Universität Bonn, 53115 Bonn, Germany
3 School of Life Sciences, Technische Universität München, 85354 Freising, Germany

* jan.hasenauer@uni-bonn.de

Contents

S1 Implementation **S3**

S2 Conserved quantities **S4**

S3 Accuracy of gradient computation **S5**

S4 Supplementary figures **S5**

References

1. Schmiester L, Schälte Y, Bergmann FT, Camba T, Dudkin E, Egert J, et al. PEtab—Interoperable specification of parameter estimation problems in systems biology. PLOS Computational Biology. 2021;17(1):1–10. doi:10.1371/journal.pcbi.1008646.

2. contributors TPBC. Benchmarking-Initiative/Benchmark-Models-PEtab: Benchmark Collection as at 2023-07-17; 2023. Available from: `https://doi.org/10.5281/zenodo.8155058`.

3. Fröhlich F, Weindl D, Schälte Y, Pathirana D, Paszkowski L, Lines GT, et al. AMICI: high-performance sensitivity analysis for large ordinary differential equation models. Bioinformatics. 2021;37(20):3676–3677. doi:10.1093/bioinformatics/btab227.

4. Fröhlich F, Weindl D, Schälte Y, Pathirana D, Paszkowski L, Lines GT, et al.. AMICI: High-Performance Sensitivity Analysis for Large Ordinary Differential Equation Models; 2024. Available from: `https://doi.org/10.5281/zenodo.10805382`.

5. Fröhlich F, Sorger PK. Fides: Reliable trust-region optimization for parameter estimation of ordinary differential equation models. PLOS Computational Biology. 2022;18(7):1–28. doi:10.1371/journal.pcbi.1010322.

6. Froehlich F, Weindl D. Fides; 2023. Available from: `https://doi.org/10.5281/zenodo.7999884`.

7. Schälte Y, Fröhlich F, Jost PJ, Vanhoefer J, Pathirana D, Stapor P, et al. pyPESTO: a modular and scalable tool for parameter estimation for dynamic models. Bioinformatics. 2023;39(11):btad711. doi:10.1093/bioinformatics/btad711.

8. Schälte Y, Fröhlich F, Stapor P, Vanhoefer J, Weindl D, Jost PJ, et al.. pyPESTO - Parameter EStimation TOolbox for python; 2024. Available from: `https://doi.org/10.5281/zenodo.10980075`.

9. Lakrisenko P, Stapor P, Grein S, Paszkowski L, Pathirana D, Fröhlich F, et al. Efficient computation of adjoint sensitivities at steady-state in ODE models of biochemical reaction networks. PLOS Computational Biology. 2023;19(1):1–19. doi:10.1371/journal.pcbi.1010783.

10. Isensee J, Kaufholz M, Knape MJ, Hasenauer J, Hammerich H, Gonczarowska-Jorge H, et al. PKA-RII subunit phosphorylation precedes activation by cAMP and regulates activity termination. Journal of Cell Biology. 2018;217(6):2167–2184. doi:10.1083/jcb.201708053.

S1 Implementation

The six test problems were taken from the PEtab benchmark collection [1, 2]. Model simulations and sensitivity computations were performed using AMICI 0.23.1 [3, 4]. Optimization was performed using the fides 0.7.8 trust-region optimizer [5, 6] via pyPESTO 0.5.0 [7, 8].

The following settings were used in AMICI to use each of the six method pairs:

$\langle \int x, \int \mathcal{J}_{\theta,\mathrm{FSA}} \rangle$

```
amici_solver.setSensitivityMethod(SensitivityMethod.forward)
amici_solver.setNewtonMaxSteps(0)
amici_model.setSteadyStateComputationMode(SteadyStateComputationMode.integrationOnly)
amici_model.setSteadyStateSensitivityMode(SteadyStateSensitivityMode.integrationOnly)
```

$\langle \int x, \int \mathcal{J}_{\theta,\mathrm{ASA}} \rangle$

```
amici_solver.setSensitivityMethod(SensitivityMethod.adjoint)
amici_solver.setNewtonMaxSteps(0)
amici_model.setSteadyStateComputationMode(SteadyStateComputationMode.integrationOnly)
amici_model.setSteadyStateSensitivityMode(SteadyStateSensitivityMode.integrationOnly)
```

$\langle \int x, \phi\, \mathcal{J}_{\theta,\mathrm{FSA}} \rangle$

```
amici_solver.setSensitivityMethod(SensitivityMethod.forward)
solver.setNewtonMaxSteps(0)
amici_model.setSteadyStateComputationMode(SteadyStateComputationMode.integrationOnly)
amici_model.setSteadyStateSensitivityMode(SteadyStateSensitivityMode.newtonOnly)
```

$\langle \int x, \phi\, \mathcal{J}_{\theta,\mathrm{ASA}} \rangle$

```
amici_solver.setSensitivityMethod(SensitivityMethod.adjoint)
amici_solver.setNewtonMaxSteps(0)
amici_model.setSteadyStateComputationMode(SteadyStateComputationMode.integrationOnly)
amici_model.setSteadyStateSensitivityMode(SteadyStateSensitivityMode.newtonOnly)
```

$\langle \phi\, x, \phi\, \mathcal{J}_{\theta,\mathrm{FSA}} \rangle$

```
amici_solver.setSensitivityMethod(SensitivityMethod.forward)
amici_solver.setNewtonMaxSteps(10000)
amici_model.setSteadyStateComputationMode(SteadyStateComputationMode.newtonOnly)
amici_model.setSteadyStateSensitivityMode(SteadyStateSensitivityMode.newtonOnly)
```

$\langle \phi\, x, \phi\, \mathcal{J}_{\theta,\mathrm{ASA}} \rangle$

```
amici_solver.setSensitivityMethod(SensitivityMethod.adjoint)
amici_solver.setNewtonMaxSteps(10000)
amici_model.setSteadyStateComputationMode(SteadyStateComputationMode.newtonOnly)
amici_model.setSteadyStateSensitivityMode(SteadyStateSensitivityMode.newtonOnly)
```

Additionally, the following problem-specific settings were used:

`Brännmark` **model**

```
amici_solver.setAbsoluteToleranceSteadyState(1.e-18)
amici_solver.setRelativeToleranceSteadyState(1.e-12)
amici_solver.setAbsoluteToleranceSteadyStateSensi(1.e-12)
amici_solver.setRelativeToleranceSteadyStateSensi(1.e-4)
```

`Fröhlich` **model**

```
amici_solver.setAbsoluteTolerance(1.e-18)
amici_solver.setRelativeTolerance(1.e-8)
amici_solver.setAbsoluteToleranceQuadratures(1.e-18)
amici_solver.setRelativeToleranceQuadratures(1.e-10)
```

`Isensee` **model**

```
amici_solver.setAbsoluteTolerance(1.e-13)
amici_solver.setRelativeTolerance(1.e-11)
amici_solver.setAbsoluteToleranceQuadratures(1.e-12)
amici_solver.setRelativeToleranceQuadratures(1.e-11)
amici_solver.setAbsoluteToleranceSteadyState(1.e-16)
amici_solver.setRelativeToleranceSteadyState(1.e-11)
```

`Weber` **model**

```
amici_solver.setAbsoluteTolerance(1.e-9)
amici_solver.setRelativeTolerance(1.e-12)
amici_solver.setAbsoluteToleranceSteadyState(1.e-9)
amici_solver.setRelativeToleranceSteadyState(1.e-12)
```

For all other settings, defaults were used.

S2 Conserved quantities

A conserved quantity is defined as a function of states that remains constant over time. Conserved quantities lead to rank-deficient Jacobians and are therefore not compatible with Newton's method or the tailored sensitivities-at-steady-state methods. The `Blasi`, `Brännmark` and `Isensee` models contained conserved quantities, which therefore, had to be removed prior to the analysis. Although not strictly necessary for $\langle \int x, \int \mathcal{J}_{\theta,\text{FSA}} \rangle$ and $\langle \int x, \int \mathcal{J}_{\theta,\text{ASA}} \rangle$, conserved quantities were removed for all six method pairs.

The conserved quantity in the `Blasi` model was removed automatically by AMICI [9]. However, the implementation in AMICI was not applicable to the `Brännmark` and `Isensee` models due to input $\mathbf{u}$ discontinuities. Therefore, for these two models the conserved quantities had to be removed manually.

Both the `Brännmark` and the `Isensee` model contained multiple conserved quantities. More specifically, for these two models total amounts of subsets of state variables remain constant

$$\sum_i x_i = \text{const} \quad \left(\text{or} \sum_i \dot{x}_i = 0 \right), \text{where}\, i \in \{1, \ldots, n_x\}.$$

Identifying each conserved quantity allows to reduce the model dimension by excluding one state variable, expressing it in terms of other state variables contained in the conserved quantity.

In the following, the removed conserved quantities are listed for both models, using identifiers from the respective SBML model.

`Brännmark` **model**

- X + Xp = const;
- IRS + IRSiP = const;
- IR + IRins + IRp + IRiP + IRi + = const.

`Isensee` **model**

- PDE + pPDE = const;
- AC + pAC + AC_Fsk + pAC_Fsk = const;
- RII_C_2 + RIIp_C_2 + RIIp_cAMP_C_2 + RIIp_cAMP_2 + RIIp_Rp8_Br_cAMPS_C_2 + RIIp_Rp8_pCPT_cAMPS_C_2 + RIIp_Rp_cAMPS_C_2 + RIIp_Sp8_Br_cAMPS_C_2 + RIIp_Sp8_Br_cAMPS_2 + RIIp_2 + RII_2 = const;
- Csub + Csub_H89 + RII_C_2 + RIIp_C_2 + RIIp_cAMP_C_2 + RIIp_Rp8_Br_cAMPS_C_2 + RIIp_Rp8_pCPT_cAMPS_C_2 + RIIp_Rp_cAMPS_C_2 + RIIp_Sp8_Br_cAMPS_C_2 = const;

Additionally, PDE, pAC and pAC_Fsk can be removed as related reactions were discarded during the analysis in [10].

S3 Accuracy of gradient computation

Similarly to [9], for pairwise comparison of steady state (objective function gradient) obtained from applying method pair 1 (MP1) and method pair 2 (MP2) we computed an error (Δ) as

$$\Delta = \begin{cases} \left|v^{\mathrm{MP2}}\right| & v^{\mathrm{MP1}} = 0 \\ \min\left(\left|v^{\mathrm{MP2}} - v^{\mathrm{MP1}}\right|, \left|\frac{v^{\mathrm{MP2}} - v^{\mathrm{MP1}}}{v^{\mathrm{MP1}}}\right|\right) & v^{\mathrm{MP1}} \neq 0 \end{cases},$$

where v^{MP1} and v^{MP2} are the steady-state (gradient values) computed with the MP1 and MP2, respectively. Used in Fig. 3, Supplementary Fig. S2, S3 and S4.

S4 Supplementary figures

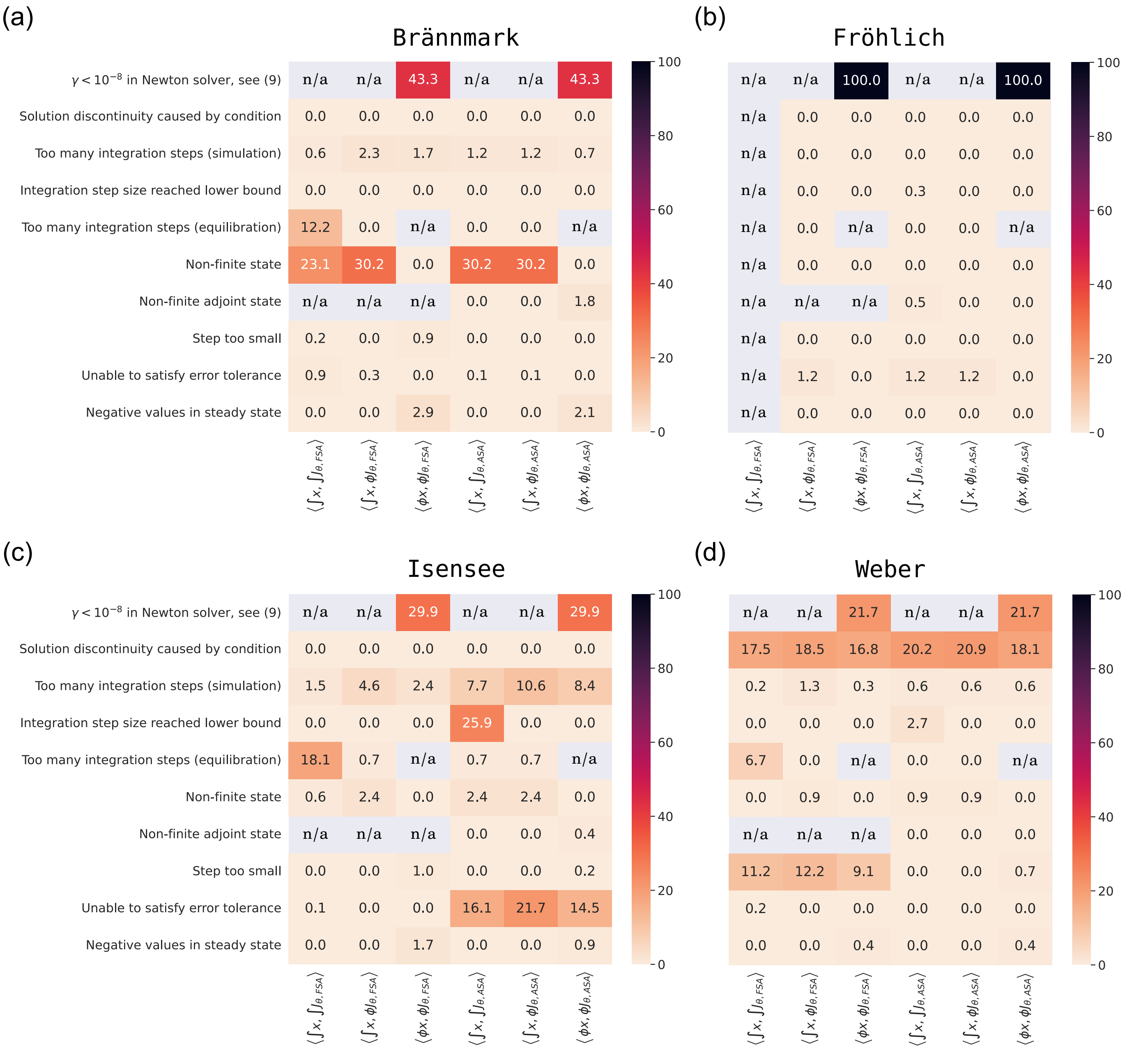

Fig S1. Failure reasons for different method pairs and problems based on 1000 simulations with randomly sampled parameter vectors. The numbers show % of total simulations. Some errors may only occur with a subset of method pairs. If an error is not possible with a method pair, it is indicated by "n/a". "Negative values in steady state" error counts the negative values that are smaller than `-100 * solver.getAbsoluteToleranceSteadyState()`. We did not encounter any failures for the `Blasi` and `Zheng` models.

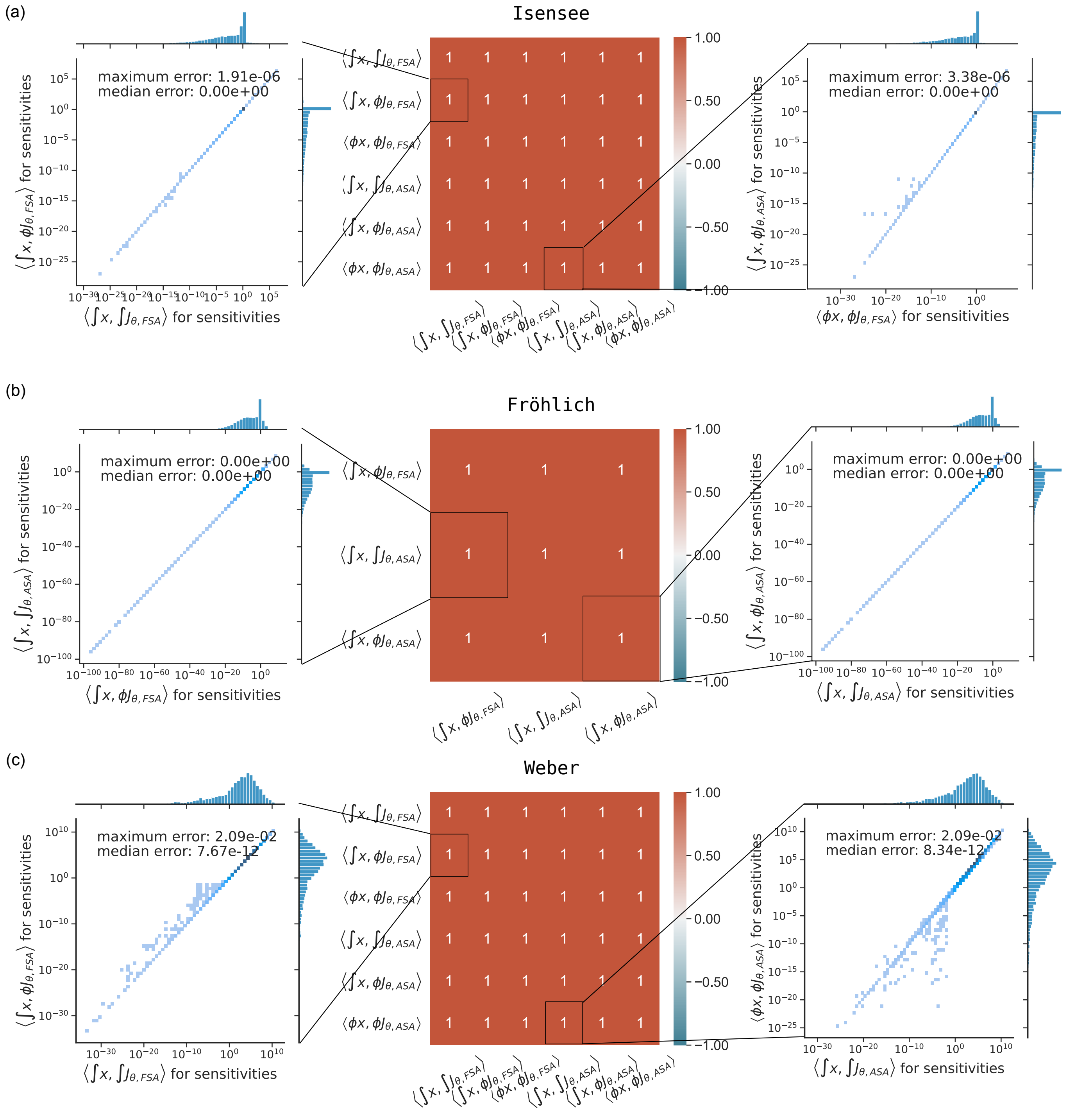

Fig S2. Comparison of steady-state values obtained from different method pairs. The heatmaps show Pearson correlation coefficients between steady-state values computed with the six different method pairs. Scatter plots visualize the difference between steady-state values for selected method pairs. Points on the diagonal indicate a good agreement, darker points indicate higher density. The maximum and median deviations were computed as defined in the supplementary section S3.

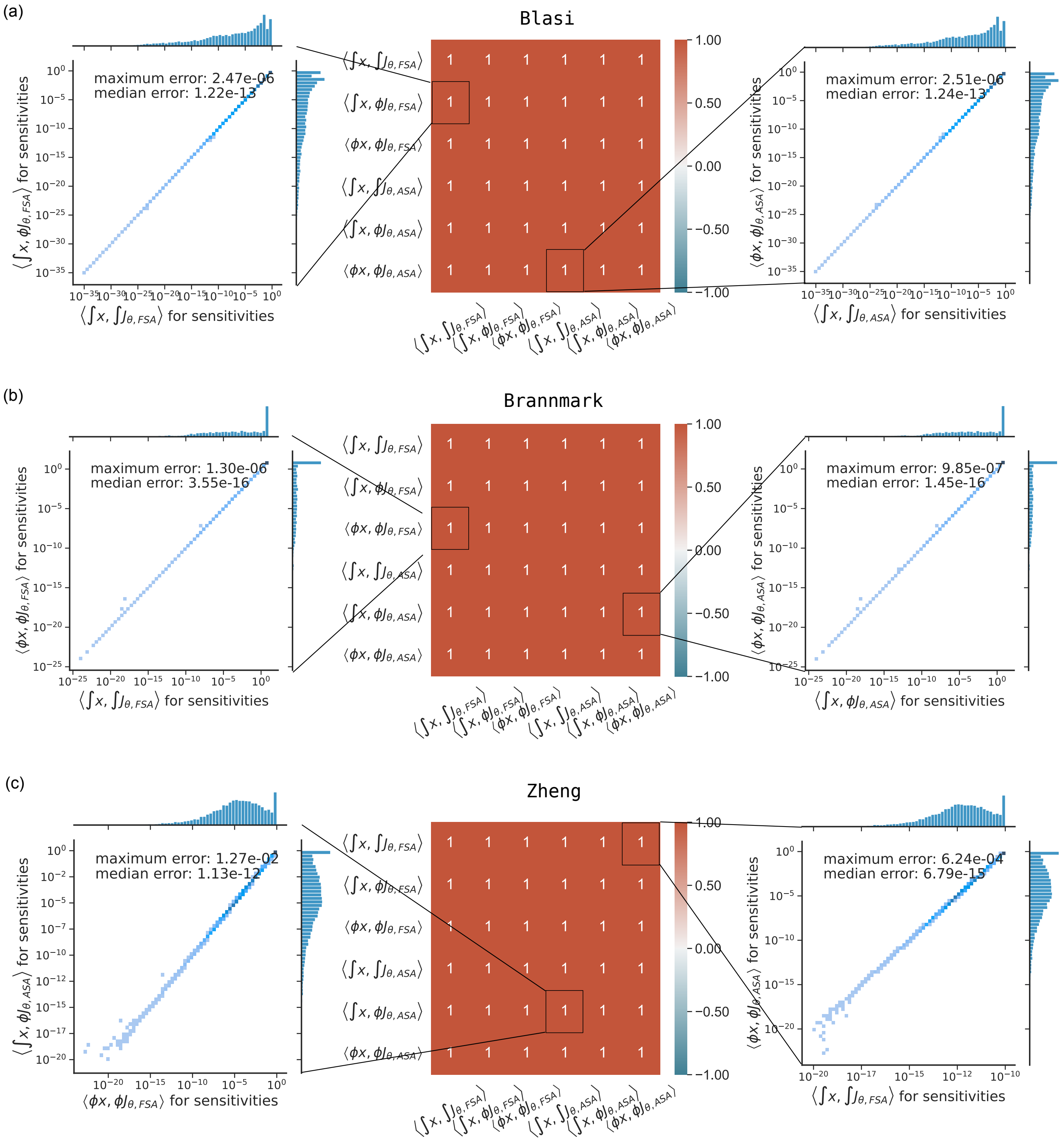

Fig S3. Comparison of steady-state values obtained from different method pairs. The heatmaps show Pearson correlation coefficients between steady-state values computed with the six different method pairs. Scatter plots visualize the difference between steady-state values for selected method pairs. Points on the diagonal indicate a good agreement, darker points indicate higher density. The maximum and median deviations were computed as defined in the supplementary section S3.

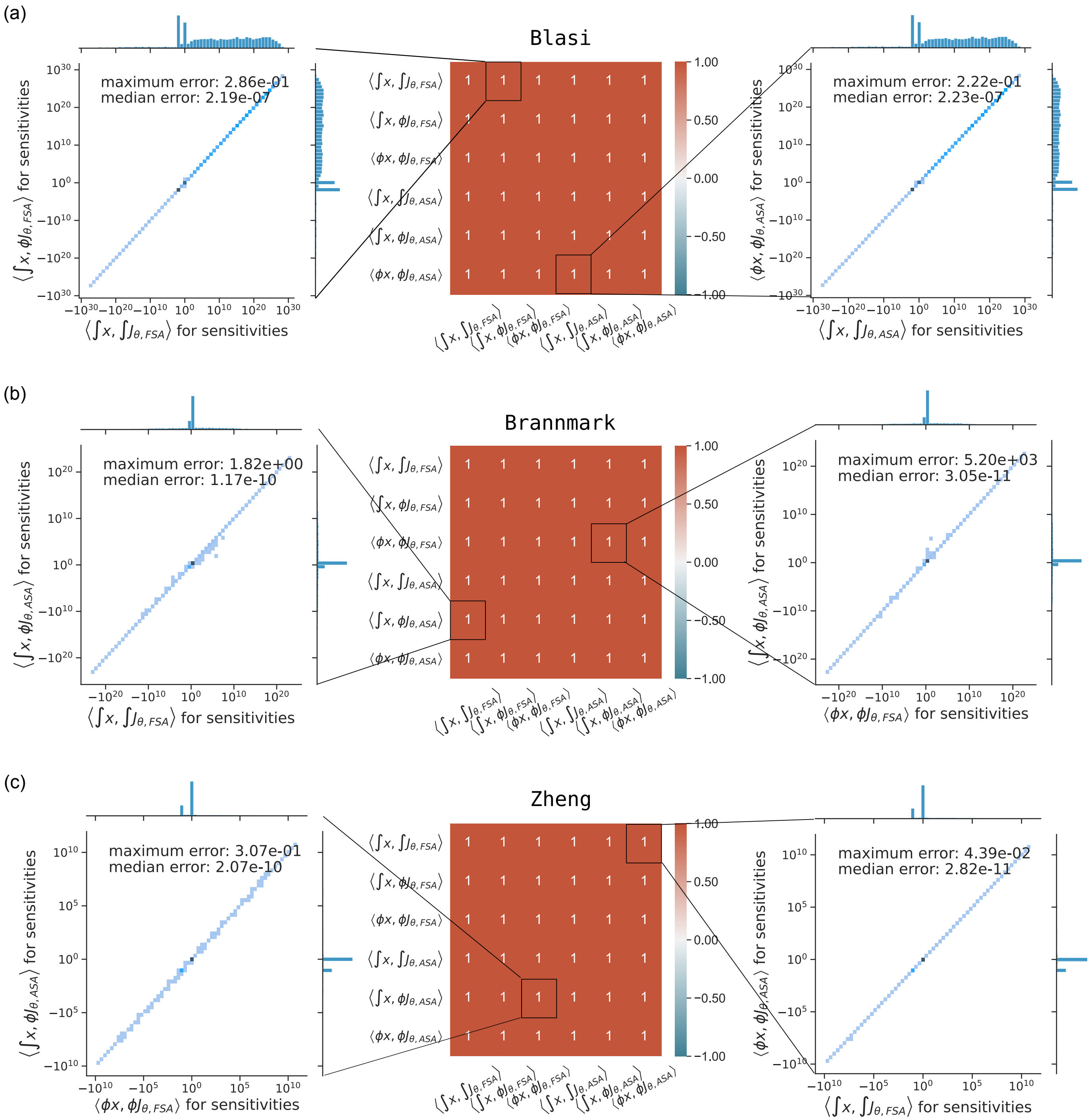

Fig S4. Comparison of objective function gradients obtained from different method pairs. The heatmaps show Pearson correlation coefficients between objective function gradient values computed with the six different method pairs. Scatter plots visualize the difference between objective function gradient values for selected method pairs. Points on the diagonal indicate a good agreement, darker points indicate higher density. The maximum and median deviations were computed as defined in the supplementary section S3.

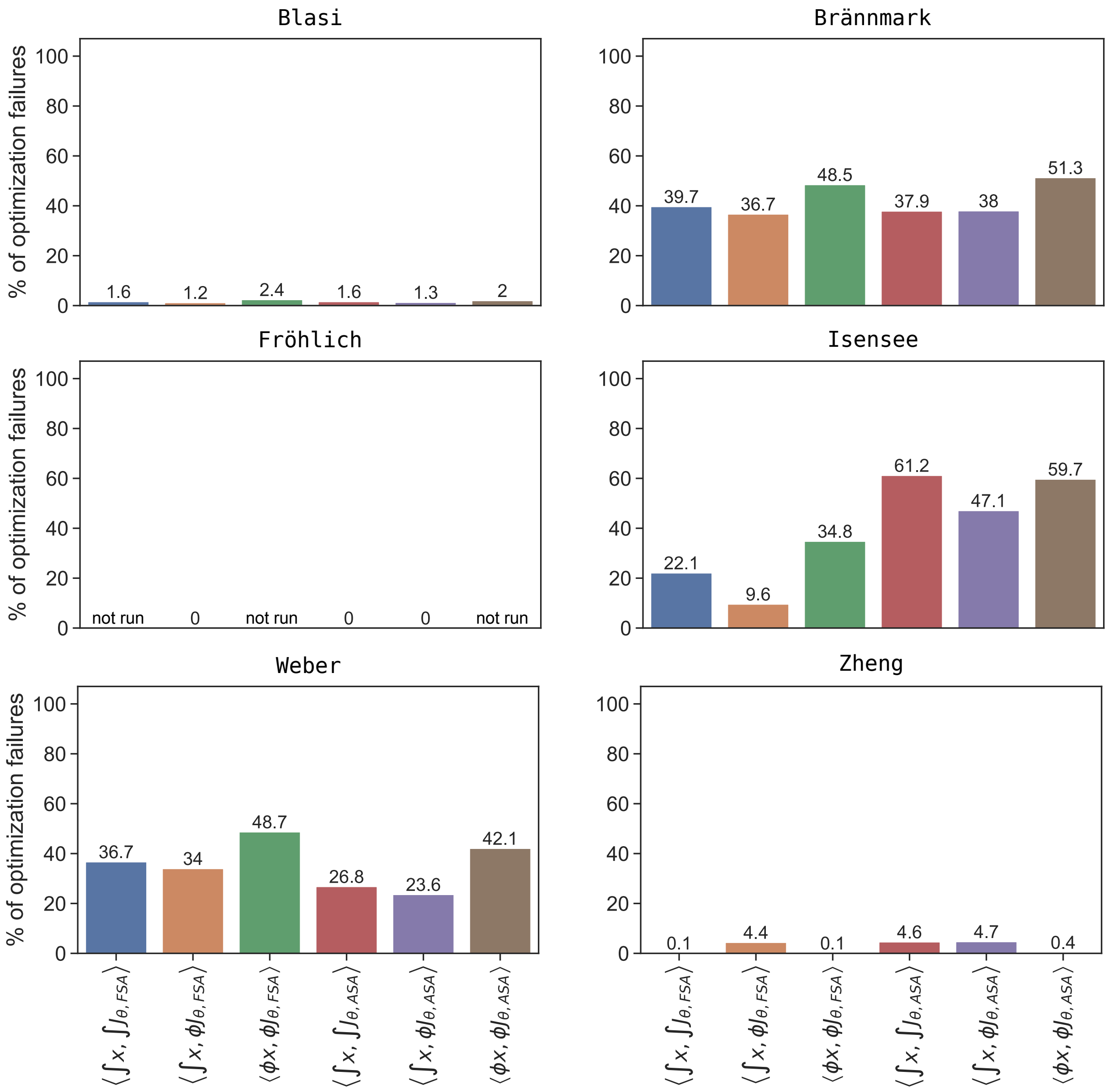

Fig S5. Failure rates for different method pairs and problems based on 1000 local optimizations initialized with randomly sampled parameter vectors.

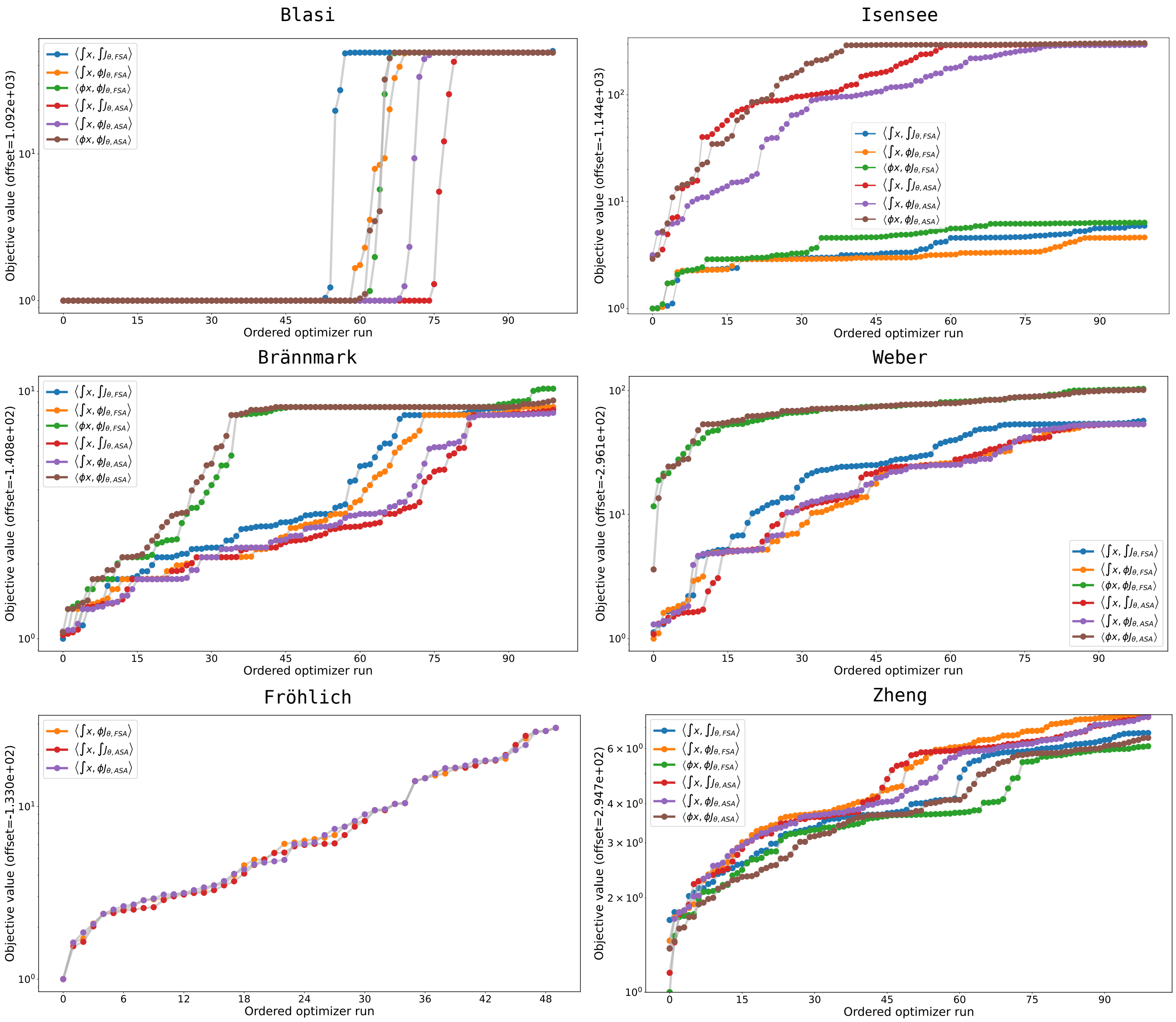

Fig S6. Comparison of final objective function values obtained from optimization when using different method pairs. For the `Blasi`, `Zheng` and `Weber` models, the 100 best objective function values are shown obtained from 1000 local optimizations. For the `Fröhlich` model, we performed 50 local optimizations with the number of optimization steps limited to 30.
