# Supplementary material for: Benchmarking methods for computing local sensitivities in ordinary differential equation models at dynamic and steady states
Source: PLoS One. 2024 Oct 23;19(10):e0312148. doi: 10.1371/journal.pone.0312148 (PMC11498742; doi:10.1371/journal.pone.0312148)
Supplement: Attachment — Submitted filename: Journal_Reviews Plos One.pdf [file pone.0312148.s002.pdf]

Review for PLOS ONE

Title: Exploration of methods for computing sensitivities in ODE models at dynamic and steady states

Dear Editor-in-Chief,

Thank you for giving me the opportunity to review for PLOS ONE. The paper presents an Exploration of methods for computing sensitivities in ODE models at dynamic and steady states. Mathematical analyses were done, and numerical computations were carried out. Kindly see the following comments and as well as suggestions for the authors:

Comments:

1. The abstract and the introduction look well written.
2. Are these 6 methods analyzed in this paper new? What is the new content in this article? The authors compared 6 different methods to solve a steady-state and sensitivities analysis for an ODE model.
3. The sentence on lines 54 to 63, does this add any new approach to science since these methods exist?
4. The Figures are blurry, therefore making the review process very difficult
5. Authors should apply these 6 methods to a given ODE model and compare the numerical results at pre-equilibration and post-equilibration
6. In conclusion, discuss your results' result/weakness/applicability. The results need to be elaborated.
