# Supplementary material for: Benchmarking methods for computing local sensitivities in ordinary differential equation models at dynamic and steady states
Source: PLoS One. 2024 Oct 23;19(10):e0312148. doi: 10.1371/journal.pone.0312148 (PMC11498742; doi:10.1371/journal.pone.0312148)
Supplement: Attachment — Submitted filename: Response to Reviewers.pdf [file pone.0312148.s003.pdf]

Dear Dr Joshua Kiddy K. Asamoah,
Dear Dr Chidozie Willaims Chukwu,
Dear Dr Emmanuel Addai,

We thank you for your constructive comments on our manuscript *“Exploration of methods for computing sensitivities in ODE models at dynamic and steady states”*. Based on your remarks, we have revised the manuscript. Our point-by-point response is provided below.

The reviewers’ comments are highlighted by italics, while the changes made in the manuscript are highlighted by yellow.

Response to Academic Editor

The manuscript has scholarly importance, and it seems very interesting. However, the title of this manuscript appears to need improvement. Try to improve your manuscript title, abstract, and introduction. The abstract should be aligned or centered. "Experimenting with methods for computing sensitivities in ordinary differential equation models" would have been fine.

We thank the Editor for the positive review and the suggestion of an alternative title. We changed the title to “Benchmarking methods for computing local sensitivities in ordinary differential equation models at dynamic and steady states”. Since those evaluated method pairs only make sense in the presence of steady-state constraints, we consider it important to include “steady state” in the title.
We also adjusted the alignment of the abstract.

Other comments:

1. *ODE should be defined as ordinary differential equation before being used.*

   We changed the title to “Benchmarking methods for computing local sensitivities in ordinary differential equation models at dynamic and steady states” and introduce the abbreviation “ODE” in the introduction:

   Changes in the manuscript:
   Initial submission:
   Many benchmark studies have been published that compare methods available for different modeling steps, such as numerical integration methods for ODE models

   Revised submission:
   Many benchmark studies have been published that compare methods available for different modeling steps, such as numerical integration methods for ordinary differential equation (ODE) models

2. *Missing punctuation mark in equation (8).*

   We thank the editor for pointing that out and added the missing comma.

3. *The authors are not consistent with a particular referencing style. Thus, the authors should ensure that they format the references according to PLOS ONE's standards.*

   We double-checked all references and ensured they have a consistent style:

   - Corrected journal names for references 1 and 16
   - Corrected DOIs for references 4, 8 and 12
   - Added volume and pages to reference 2
   - Fixed capitalization in the titles for references 4, 5, 13, 15, 16, 17, 19, 20, 22, 23, 29, 31 and 32

4. *Are the figures part of the main work? If they are, then they should be included in the main manuscript and should be explicated. They should be given figure numbers. The first pdf figures is not clear.*

   We apologise for the inconvenience. However, PLOS ONE submission guidelines require that figures are removed from the main text (https://journals.plos.org/plosone/s/latex#loc-figures). We received this message "Please remove your figures from within your manuscript file, leaving only the individual TIFF/EPS image files. These will be automatically included in the reviewer's PDF.", and had to resubmit the manuscript without the figures. We agree that this is not convenient, but unfortunately, we cannot address the issue.

5. *Authors should apply these 6 methods to a given ODE model and compare the numerical results at pre-equilibration and post-equilibration.*

   All sensitivity methods analysed in our manuscript have been introduced before, with the most recent one being the tailored ASA approach (doi:10.1371/journal.pcbi.1010783). However, the best choice is often not obvious and many researchers might not even be aware of the available methods. Therefore, we compiled this comprehensive overview and evaluated all those different methods on a set of real-world problems, which has not been done before.

   Changes in the manuscript:

   Initial submission:
   In this study we explored six combinations of methods for computing steady states and sensitivities at steady state, and evaluated their accuracy, efficiency and robustness based on six real-world problems.

Revised submission:
In this study, we explored six combinations of methods for computing steady states and sensitivities at steady state, and evaluated their accuracy, efficiency and robustness based on six real-world problems. All those methods have been introduced before, but have not yet been evaluated on a joint set of problems.

6. *What is the difference between this current work and " PeTTSy: a computational tool for perturbation analysis of complex systems biology models" and Sensitivity analysis of infectious disease models: methods, advances and their application*

   The referenced manuscripts (“PeTTSy: a computational tool for perturbation analysis of complex systems biology models" by Domijan et al.; and “Sensitivity analysis of infectious disease models: methods, advances and their application” by Wu et al.) use rather different methods and have different objectives than our work: Both Domijan et al. and Wu et al. are interested in global sensitivities and analyse them using various qualitative and quantitative methods. However, in our work, we are interested in local sensitivities (i.e., the change of some output value in response to an infinitesimal change in some input value around a reference point) and how their computation affects parameter estimation using gradient-based optimization. For non-linear models, local and global sensitivities generally differ. For gradient-based optimization, local sensitivities are required. Therefore, none of the methods employed by either Domijan et al. or Wu et al. are suitable in this context. Moreover, we focus on the cases that involve steady-state computation and include in our analysis methods that are tailored to this situation in order to speed up the computations. Neither Domijan et al. nor Wu et al. consider steady-state computation, and neither perform a quantitative comparison of robustness or efficiency of the different methods.

   We updated the title and the introduction to clarify that our manuscript is only concerned with local sensitivities:

   Changes in the manuscript:
   Initial title:
   Exploration of methods for computing sensitivities in ODE models at dynamic and steady states
   Revised title:
   Benchmarking methods for computing local sensitivities in ordinary differential equation models at dynamic and steady states

   Initial submission:
   The most widely used approaches for computing sensitivities include finite differences, automatic differentiation, forward sensitivity analysis (FSA), and adjoint sensitivity analysis (ASA).

   Revised submission:

The most widely used approaches for computing local sensitivities include finite differences, automatic differentiation, forward sensitivity analysis (FSA), and adjoint sensitivity analysis (ASA).

Response to Reviewer 1

1. *The abstract and the introduction look well written.*

   We thank the reviewer for the positive review.

2. *Are these 6 methods analyzed in this paper new? What is the new content in this article? The authors compared 6 different methods to solve a steady-state and sensitivities analysis for an ODE model.*

   All sensitivity methods analysed in our manuscript have been introduced before, with the most recent one being the tailored ASA approach (doi:10.1371/journal.pcbi.1010783). However, the best choice is often not obvious and many researchers might not even be aware of the available methods. Therefore, we compiled this comprehensive overview and evaluated all those different methods on a set of real-world problems, which has not been done before.

   Changes in the manuscript:

   Initial submission:
   In this study we explored six combinations of methods for computing steady states and sensitivities at steady state, and evaluated their accuracy, efficiency and robustness based on six real-world problems.

   Revised submission:
   In this study, we explored six combinations of methods for computing steady states and sensitivities at steady state, and evaluated their accuracy, efficiency and robustness based on six real-world problems. All those methods have been introduced before, but have not yet been evaluated on a joint set of problems.

3. *The sentence on lines 54 to 63, does this add any new approach to science since these methods exist?*

   Reviewer 1 is right in that all applied methods already existed and that this manuscript does not introduce any new methodology. However, we consider this evaluation and comparison of those different methods a valuable contribution to make researchers aware of the different methods and point out potential problems. This will help developers of simulation tools to prioritise the implementation of the different method pairs and help users to choose from the available methods.

4. *The Figures are blurry, therefore making the review process very difficult*

   We apologise for the inconvenience. These figures were automatically added to the pdf during the submission process. It is possible to download a high-resolution version of each figure by clicking the black arrow at the top of each preview page.
   The submission guidelines state: “The compiled submission PDF includes low-resolution preview images of the figures after the reference list. The function of these previews is to allow you to download the entire submission as quickly as possible.”.

5. *Authors should apply these 6 methods to a given ODE model and compare the numerical results at pre-equilibration and post-equilibration*

   We applied those six methods to six different ODE models from real-world problems. Whereas in the main text we only show the comparison of the objective function, supplementary figures S1 and S2 show the requested comparison of the steady states computed during pre- or post-equilibration. These data indicate good agreement across the different methods up to chosen integration tolerances (given in supplementary section 1). These figures are referenced in the result section, line 260.

6. *In conclusion, discuss your results’ result/weakness/applicability. The results need to be elaborated.*

   We thank the reviewer for bringing this to our attention. We have now given this greater emphasis in the manuscript.

   Changes in the manuscript:
   In this study, we explored six combinations of methods for computing steady states and sensitivities at steady state and evaluated their accuracy, efficiency and robustness based on six real-world problems. All those methods have been introduced before, but have not yet been evaluated on a joint set of problems. The considered methods are widely applicable and do not require any specific model properties. However, it should be taken into account that the standard FSA approach is the least scalable and becomes prohibitively computationally expensive for large-scale models, while the more scalable ASA approach avoids computation of state sensitivities that may be needed for further analysis.

   While we would like to derive general guidelines for choosing the best method for a given model, we have to note that our results might not generalize fully. Our study showed that the impact of the explored approaches on failure rates and speedup are quite problem-dependent. We based our analysis on six of the seven models available in the PEtab benchmark collection [9]. Further extension of the model collection, in particular by models that require steady-state computation, would allow for a more comprehensive analysis.

Response to Reviewer 2

The study explore six method pairs for computing the steady state and sensitivities at steady state using six real-world problems. The method pairs involve numerical integration or Newton's method to compute the steady-state, and – for both forward and adjoint sensitivity analysis–numerical integration or a tailored method to compute the sensitivities at steady-state. The paper is potentially interesting and useful. However, the author needs to pay attention to the following suggestions:

1. *The abstract general overview is too long and lacks specific results that highlight the main findings. It would be beneficial to include a sentence summarising the key outcomes of this study.*

   We shortened the abstract as suggested by Reviewer 2 and expanded the summary of the key outcomes:

   Changes in the manuscript:
   Estimating parameters of dynamic models from experimental data is a challenging, and often computationally-demanding task. It requires a large number of model simulations and objective function gradient computations, if gradient-based optimization is used. ~~The gradient depends on derivatives of the state variables with respect to parameters, also called state sensitivities, which are expensive to compute.~~
   In many cases, steady-state computation is a part of model simulation, either due to steady-state data or an assumption that the system is at steady state at the initial time point. Various methods are available for steady-state and gradient computation.
   Yet, the most efficient pair of methods (one for steady states, one for gradients) for a particular problem is often not clear. ~~Moreover, depending on the model and the available data, some methods may not be applicable or sufficiently robust.~~ In order to facilitate the selection of methods, we explore six method pairs for computing the steady state and sensitivities at steady state using six real-world problems, and evaluate their accuracy, efficiency and robustness. The method pairs involve numerical integration or Newton's method to compute the steady-state, and -- for both forward and adjoint sensitivity analysis -- numerical integration or a tailored method to compute the sensitivities at steady-state.
   Our evaluation shows that all method pairs provide accurate steady-state and gradient values, and that the two method pairs that combine numerical integration for the steady-state with a tailored method for the sensitivities at steady-state were the most robust, and amongst the most computationally-efficient. We also observed that while Newton's method for steady-state computation yields a substantial speedup compared to numerical integration, it led to a large number of simulation failures. Overall, our study provides a concise overview across current methods for computing sensitivities at steady

state. While our study shows that there is no universally-best method pair, it also provides guidance to modelers in choosing the right methods for a problem at hand.

2. *Consider increasing the visibility of the figures and give proper citation for each in the discussion.*

   We had to remove the figures from the main text following PLOS ONE submission guidelines (https://journals.plos.org/plosone/s/latex#loc-figures). Only figure captions remained in the text, e.g. the top of the page 9. In the final publication, the figures will be placed above the captions. Unfortunately, the (required) separate submission of the figures led to them being included in poor quality by the submission system. A high-resolution version of the figures can be downloaded by clicking the black arrow at the top of each figure preview page.
   All of the figures are referenced in the results section.

3. *Provide more detailed explanations of failure cases, particularly for methods that showed high failure rates (e.g., Newton's method for the Fröhlich model).*

   We added more details on the modes of failure to the main text and added an additional supplementary figure.

   New supplementary Figure S1:

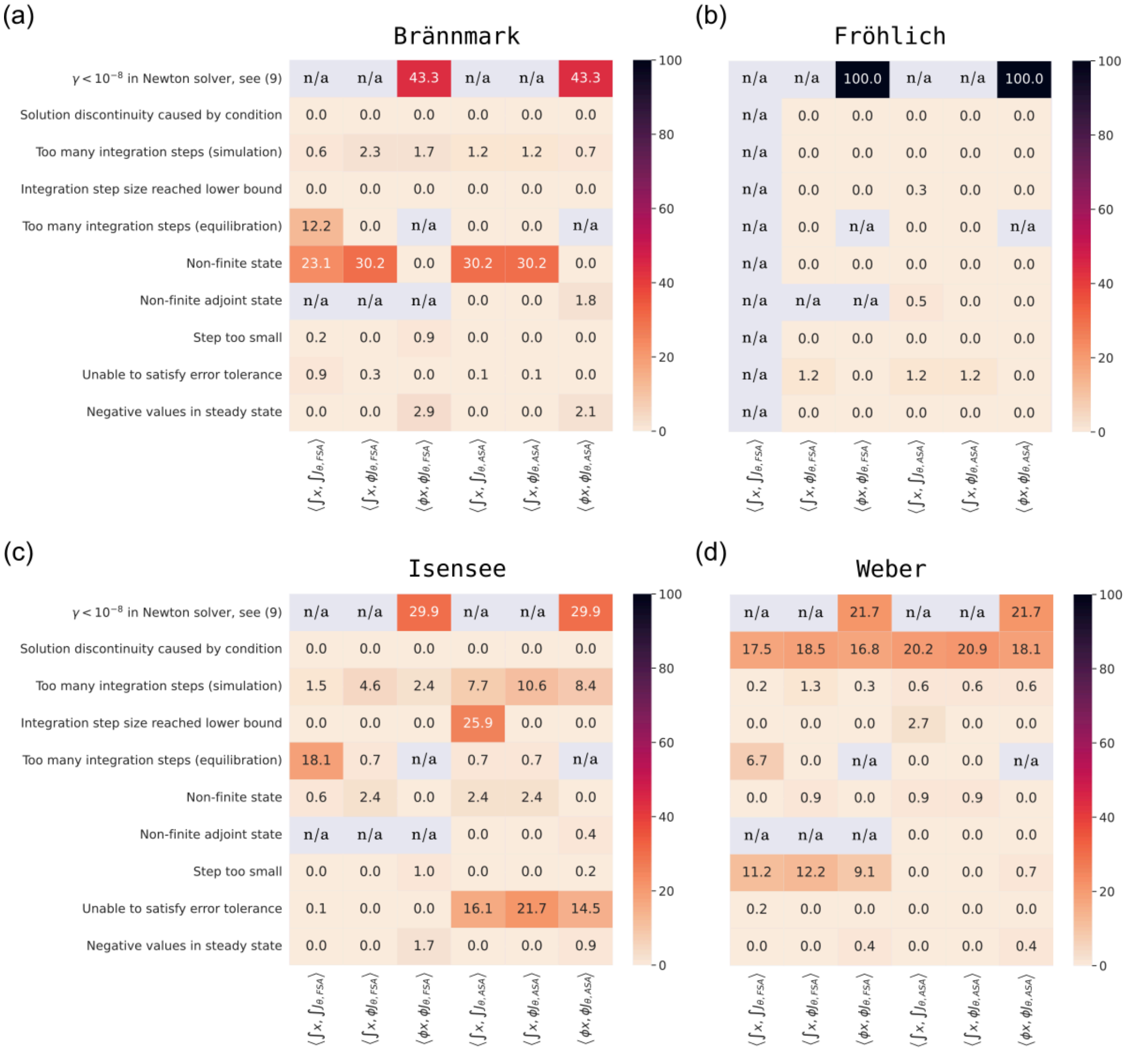

Figure S1: **Failure reasons for different method pairs and problems based on 1000 simulations with randomly sampled parameter vectors.** The numbers show % of total simulations. Some errors may only occur with a subset of method pairs. If an error is not possible with a method pair, it is indicated by "n/a". "Negative values in steady state" error counts the negative values that are smaller than `-100 * solver.getAbsoluteToleranceSteadyState()`. We did not encounter any failures for the Blasi and Zheng models.

Changes in the manuscript:

Initial submission:

For the Brännmark, Isensee and Weber models, the failure rate for the six method pairs ranged from 31.5% with… to 48.8% with …, from 7.7% with … to 54.3% with …, and from 22.4% with … to 48.3% with…, respectively. For the Froehlich model, …

Revised submission:

For the Brännmark, Isensee and Weber models, the failure rate for the six method pairs ranged from 31.5% with… to 48.8% with …, from 7.7% with … to 54.3% with …, and from 22.4% with … to 48.3% with…, respectively. The experimental conditions for the Weber model are described using a discontinuous function, which caused numerical problems in about 17% of the simulations for each of the six method pairs (Fig. S1). With the Brännmark model, we observed that for about 30% of sampled parameters simulations failed independently of the applied method (Fig. S1). For the Fröhlich model, […]

Initial submission:

The reason for the high failure rate with Newton's method varied between models. For the Fröhlich model, Newton's method failed with every parameter vector due to numerical errors in at least one of the n_u steady-state computations.

Revised submission:

The reason for the high failure rate with Newton's method varied between models. The most common reason for all models is that the factor γ in (9) reached a lower bound while the condition (8) was not fulfilled, i.e. the steady state could not be computed because the initial guess was too far away from it, and even the modification described in (9) could not resolve the issue. For the Froehlich model, Newton's method failed with every parameter vector due to numerical errors in at least one of the n_u steady-state computations. [...]

The hypothesis that the failure rate for Newton's method depends on the number of state variables could not be confirmed based on the given set of problems --- both the Fröhlich model with a large number of state variables and the Brännmark model with very few state variables exhibited high failure rates.
